# Pilomatrixcarcinoma of the Foot: A New Localization of an Extremely Rare Adnexal Tumour

**DOI:** 10.3390/diagnostics11050793

**Published:** 2021-04-28

**Authors:** Gerardo Cazzato, Anna Colagrande, Paolo Romita, Caterina Foti, Leonardo Resta, Giuseppe Ingravallo

**Affiliations:** 1 Section of Pathology, Department of Emergency and Organ Transplantation, University of Bari, 70124 Bari, Italy; annacolagrande@gmail.com (A.C.); leonardo.resta@uniba.it (L.R.); giuseppe.ingravallo@uniba.it (G.I.); 2 Department of Biomedical Science and Human Oncology, Dermatological Clinic, University of Bari, 70124 Bari, Italy; romitapaolo@gmail.com (P.R.); caterina.foti@uniba.it (C.F.)

**Keywords:** pilomatrixcarcinoma, toe, adnexal tumour

## Abstract

Pilomatrixcarcinoma is a very rare follicular neoplasm that shows matrical differentiation. The majority of these lesions originate de novo, while only a few cases of transformation of pilomatricoma (calcifying epithelioma of Malherbe) have been described in the literature. The neoplasm affects mostly middle-aged males with a male-to-female ratio of 3–4:1. The most common localizations are the face, head, trunk and extremites, though there are a few reports of pilomatrixcarcinoma of the eyelid, eyebrow, axilla and clitorid. Here, we describe the first case of a pilomatrixcarcinoma on the anterolateral surface of the first toe of the left foot of an 83-year-old patient, which developed in less than six months and led to amputation of the distal phalanx. We report a brief review of the current literature with particular emphasis on histopathological features useful for diagnosis.

An 83-year-old man was referred to our hospital due to the onset of a grayish nodule that appeared a few months earlier on the anterolateral surface of the first toe of the left foot in the perionichial area. The patient had no significant history of skin cancer and was undergoing therapy for essential arterial hypertension. The laboratory tests showed only a slight increase in the Erythrocyte sedimentation rate (VES), with all other parameters completely normal. 

The dermoscopic study of the lesion revealed a solitary nodule, about 3.0 cm in diameter, and on palpation, it appeared as a firm nodule. After a standard radiograph was performed, it was decided to subject the patient to an incisional biopsy to ascertain the nature of the lesion. The diagnosis of adnexal-type carcinoma was made, even if the intrinsic characteristics of the biopsy did not allow a nosographic diagnosis of certainty. The patient then underwent a distal phalangectomy with grafting of a skin flap to close the skin break.

Gross examination of the sample confirmed the presence of an exophytic-polypoid lesion that seemed to infiltrate the deep plane (Figure 1A). A section conducted along the median sagittal plane (Figure 1B) confirmed this impression, with a lesion of about 2.4 cm, which extensively infiltrated the subcutaneous tissue until it approached the bone plane.

Gross specimen: an exophytic lesion is obvious on the anterolateral surface of the first toe (*) (Figure 1A); the surface section along the median sagittal plane shows a lesion of about 2.4 cm extended to the subcutaneous tissue (Figure 1B—included within the red line).

After sampling, processing, paraffin-embedding and microtome-cutting, five-micron-thick sections were obtained and stained with routine staining (Hematoxylin-Eosin). Histologically, lesions appeared as a poorly circumscribed and asymmetric neoplasm, without ulceration of overlying epidermis (Figure 1C). Neoplasm consisted of a proliferation of immature basaloid cells, grouped together in solid aggregates, sometimes in the form of cords of neoplastic cells with rare “ghost cells” involving the deep dermis, and subcutaneous tissue (Figure 2A–C). The basaloid matrical cells forming the epithelial component of the neoplasm were elongated, with a variable amount of cytoplasm, vescicular nucleus and a prominent nucleolus (Figure 2C). There was a very high number of atypical mitoses, but very few shadow cells compared to pilomatricoma. Necrosis “en masse” and multinucleated histyocites were prominent, with scant stroma and abundant vascularization.

To ensure that it was an adnexal lesion, an immunohistochemical reaction was carried out for Beta-catenin, which was found to be positive mainly at the level of the membrane of the matrix cells but also weakly present at the level of the cytoplasm (Figure 2D). 

Diagnosis of Pilomatrixcarcinoma was made.

About 135 cases of Pilomatrixcarcinoma were been published in the English language literature [1]. Among these, the most frequent localizations were represented by the head, face, trunk and upper extremities [1,2,3,4,5,6]. Rare and anecdotal cases have been described in locations such as neck, eyelid, eyebrown, axilla and clitoris [2,3,4,5]. Pilomatrixcarcinoma has never been described in the toes: ours is the first case described in the literature, in a patient who developed the lesion in about five and a half months.

The clinical suspicion was squamous carcinoma, although this was not completely in accordance with the rapid timing of onset, more typical of malignant adnexal neoplasms [5,6]. Histopathologic differential diagnosis of pilomatrixcarcinoma includes pilomatricoma and basal cell carcinoma with matrical differenziation, while immunohistochemistry is accessory, as the diagnosis is purely histological.

Some authors have described [6,7] nuclear and cytoplasmic staining for beta-catenin in Pilomatrixcarcinoma and, furthermore, in a few cases studied by molecular biology, mutations in the beta-catenin gene CTNNB1 have been found [8]. A higher incidence of malignant adnexal neoplasms in immunocompromised subjects has also been described [9], but this underlying condition was not present in our case.

We briefly described a new onset site of pilomatric carcinoma of the first toe of an elderly patient, with no apparent signs of immunocompromise. A careful histopathological evaluation and integration of clinical-anamnestic data, together with the knowledge of this entity, are fundamental prerequisites for a better outcome for the patient.

## Figures and Tables

**Figure 1:**
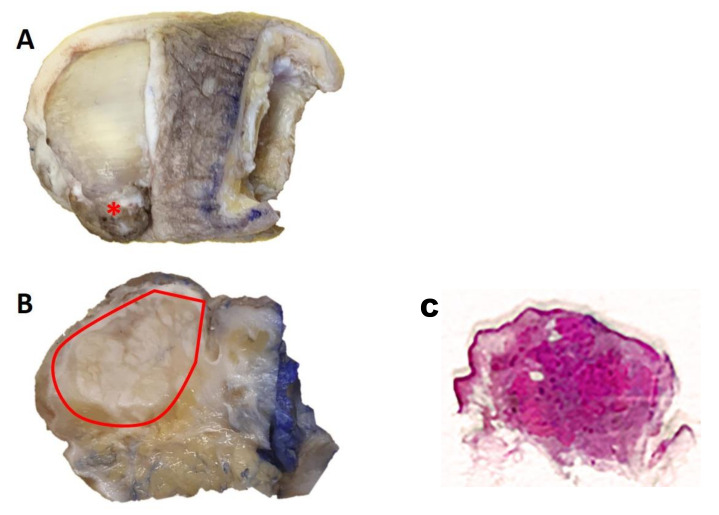
(**A**) Gross examination of the sample confirmed the presence of an exophytic-polypoid lesion that seemed to infiltrate the deep plane; (**B**) A section conducted along the median sagittal plane; (**C**) Histologically, lesions appeared as a poorly circumscribed and asymmetric neoplasm, without ulceration of overlying epidermis.

**Figure 2:**
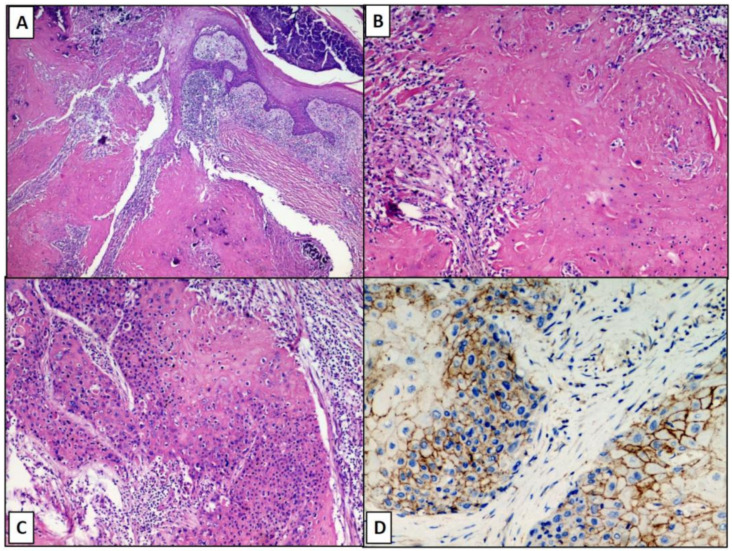
Pilomatrixcarcinoma appears as a poorly circumscribed and asymmetric neoplasm, without ulceration of overlying epidermis and with necrotic areas (original magnification 10×; **A**); Detail of necrotic areas with the focal presence of shadow cells (original magnificaton 20×; **B**); basaloid cells surrounding the areas with necrosis en masse (original magnification 20×, **C**); predominantly membrane positivity (but also weakly cytoplasmic) for Beta-catenin at the level of matrical cells (IHC, Beta-catenin antibody, 40×, **D**).

## Data Availability

The data presented in this study are available on request from the corresponding author.
